# AI driven quantitative analysis of meibomian glands in children and adolescents: a benchmark dataset study

**DOI:** 10.1186/s40662-025-00460-2

**Published:** 2025-11-06

**Authors:** Li Li, Kunhong Xiao, Kunfeng Lai, Taichen Lai, Yujie Wang, Xianwen Shang, Ying Xue, Zongyuan Ge, Lingyi Liang, Mingguang He, Jiawen Lin, Zhuoting Zhu

**Affiliations:** 1 Center for Eye Research Australia, Royal Victorian Eye and Ear Hospital, East Melbourne, Australia; 2 Department of Surgery (Ophthalmology), The University of Melbourne, Melbourne, Australia; 3 Department of Ophthalmology, Shengli Clinical Medical College of Fujian Medical University, Fuzhou University Affiliated Provincial Hospital, Fuzhou, China; 4 Department of Ophthalmology and Optometry, Fujian Medical University, Fuzhou, China; 5 School of Computer Science and Big Data, Fuzhou University, Fuzhou, China; 6 Monash E-Research Center, Monash University, Melbourne, VIC Australia; 7 Monash Medical AI Group, Monash University, Melbourne, VIC Australia; 8 State Key Laboratory of Ophthalmology, Zhongshan Ophthalmic Center, Sun Yat-Sen University, Guangdong Provincial Key Laboratory of Ophthalmology and Visual Science, Guangzhou, China; 9 School of Optometry, The Hong Kong Polytechnic University, Hong Kong, China; 10 Research Center for SHARP Vision, The Hong Kong Polytechnic University, Kowloon, Hong Kong SAR China; 11 Fujian Key Laboratory of Network Computing and Intelligent Information Processing, Fuzhou University, Fuzhou, China

**Keywords:** Meibomian gland, Artificial intelligence, Children, Adolescents, Dry eye disease

## Abstract

**Background:**

Due to the lack of quality-controlled quantitative data on meibomian gland (MG) morphology in children and adolescents, this study aims to establish a Children and Adolescents Meibomian Gland (CAMG) dataset.

**Methods:**

A total of 1114 quality-controlled upper eyelid infrared images were collected from 730 children and adolescent subjects using the Oculus Keratograph 5 M. Images underwent preprocessing and multi-stage expert quality control screening before segmentation. Morphological parameters including gland area, gland dropout ratio, gland length and width, number of glands, and total glands ratio were extracted using an AI model. The dataset, comprising images, annotations, and demographic information, is openly accessible on Figshare, with AI model codes available on GitHub to support research reproducibility and algorithm optimization.

**Results:**

The dataset includes 1114 high-resolution quality-controlled upper eyelid images from 730 subjects (mean age 11.80 ± 2.39 years; 46.77% male), accompanied by AI-assisted segmentation annotations and corresponding morphological measurements. The U-Net segmentation model achieved an accuracy of 97.49%, a Dice coefficient of 89.72%, and an intersection over union (IoU) of 81.67%. Quantitative analysis revealed that MG parameters remained relatively stable in adolescents compared to children. Females exhibited significantly wider and larger MGs than males. Similar sex-related differences were also observed in the central five MGs. Males exhibited a higher MG count compared to females.

**Conclusions:**

CAMG is a publicly available MG dataset for children and adolescents to support AI-based individualized clinical assessments. The dataset's transparent quality control processes establish a foundation for epidemiological research, promoting cross-institutional collaboration and AI-driven advancements in ophthalmology.

**Supplementary Information:**

The online version contains supplementary material available at 10.1186/s40662-025-00460-2.

## Background

Dry eye disease is one of the most common reasons for ophthalmology clinic visits and significantly affects patients' work efficiency and quality of life [1]. The MGs are responsible for secreting the lipid layer of the tear film and dysfunction of these glands can lead to tear film instability, triggering or worsening dry eye symptoms [2]. With the widespread adoption of infrared imaging, meibography has emerged as a critical tool for clinical evaluation of gland morphology [3]. However, current interpretations of meibography remain heavily reliant on clinicians' subjective expertise, leading to significant diagnostic variability [4]. This manual assessment not only hinders standardization but also contributes to inconsistent conclusions across healthcare institutions.

Artificial intelligence (AI) breakthroughs in medical image analysis offer transformative solutions [5]. In the evaluation of MGs, a range of traditional computer vision techniques such as the watershed algorithm [6], Gabor wavelets [7], the Riesz pyramid [8], and global Fourier transform [9, 10] have been successfully employed for image processing and feature extraction. Machine learning approaches, including convolutional neural networks (CNN) [11–14], generative adversarial networks (GAN) [15], and vision transformer (ViT) [16] have also shown considerable promise in automating the analysis of glandular structures, improving the precision of diagnosis, and facilitating large-scale assessments with minimal human intervention. However, the clinical translation of these AI systems faces significant challenges related to training data quality. Previous studies have demonstrated substantial inter-observer variability in meibography analysis, with examiners showing inconsistent delineation of gland boundaries and tarsus margins [17–19]. These manual annotation discrepancies present multifaceted challenges for AI model development: (1) inconsistent ground truth labels during training can lead to conflicting learning signals, reducing model convergence and accuracy; (2) evaluation becomes unreliable when reference standards vary between annotators; and (3) models trained on inconsistent annotations may exhibit poor generalization across different clinical settings or examiners. Such annotation variability necessitates robust training strategies and quality control measures to ensure reliable AI-assisted meibography analysis.

Notably, the incidence of MG atrophy in children and adolescents has risen sharply in recent years [20]. However, research in this population remains limited, largely due to challenges in acquiring high-quality imaging and ensuring examination compliance in younger age groups. To address this gap, we developed the Children and Adolescents Meibomian Gland (CAMG) dataset—an open-access, large-scale resource comprising quality-controlled upper eyelid infrared images from individuals aged 4–18 years. Following the World Health Organization (WHO) definitions [21], we classified participants aged 4–17 years as children and those aged 10–18 years as adolescents, acknowledging that the two categories partially overlap. The dataset includes expert-validated AI segmentations and quantitative morphological parameters to support age-specific analysis of meibomian gland development. This study focused exclusively on upper eyelid meibography for several reasons: (1) the upper eyelid contains approximately 61% of total MGs, representing the majority of glandular tissue [22]; (2) upper eyelid glands demonstrate greater morphological variability and are more prone to pathological changes such as tortuosity and hooking [23]; (3) clinical symptoms of dry eye disease correlate more strongly with upper eyelid gland dysfunction [24]; and (4) in the pediatric population, lower eyelid imaging often yields suboptimal image quality due to patient cooperation challenges.

The CAMG dataset provides a foundational reference for characterizing normal MG development across pediatric age ranges, enabling individualized, age-matched assessments of gland health. This benchmark data supports early identification of MG dysfunction and contributes to more accurate diagnosis and risk stratification of pediatric dry eye disease. Additionally, the dataset offers a valuable basis for epidemiological studies examining the influence of environmental exposures, such as digital screen time, on gland morphology. By promoting data transparency and research reproducibility, CAMG facilitates the development and validation of AI algorithms and contributes to the standardization of ocular surface disease assessment in young populations.

## Methods

### Data collection

MG images were captured using the Keratograph 5 M (Oculus, Germany), with all images obtained by the same trained technician to ensure consistency across the dataset. The inclusion criteria included children and adolescents aged 4–18 years, with no history of ocular surface diseases, contact lenses wear or systemic conditions affecting the MGs. Images showing reflections, incomplete visualization of the MGs, or blurred gland structures were excluded from the dataset to maintain the standard of data required for subsequent analysis.

**Fig. 1:**
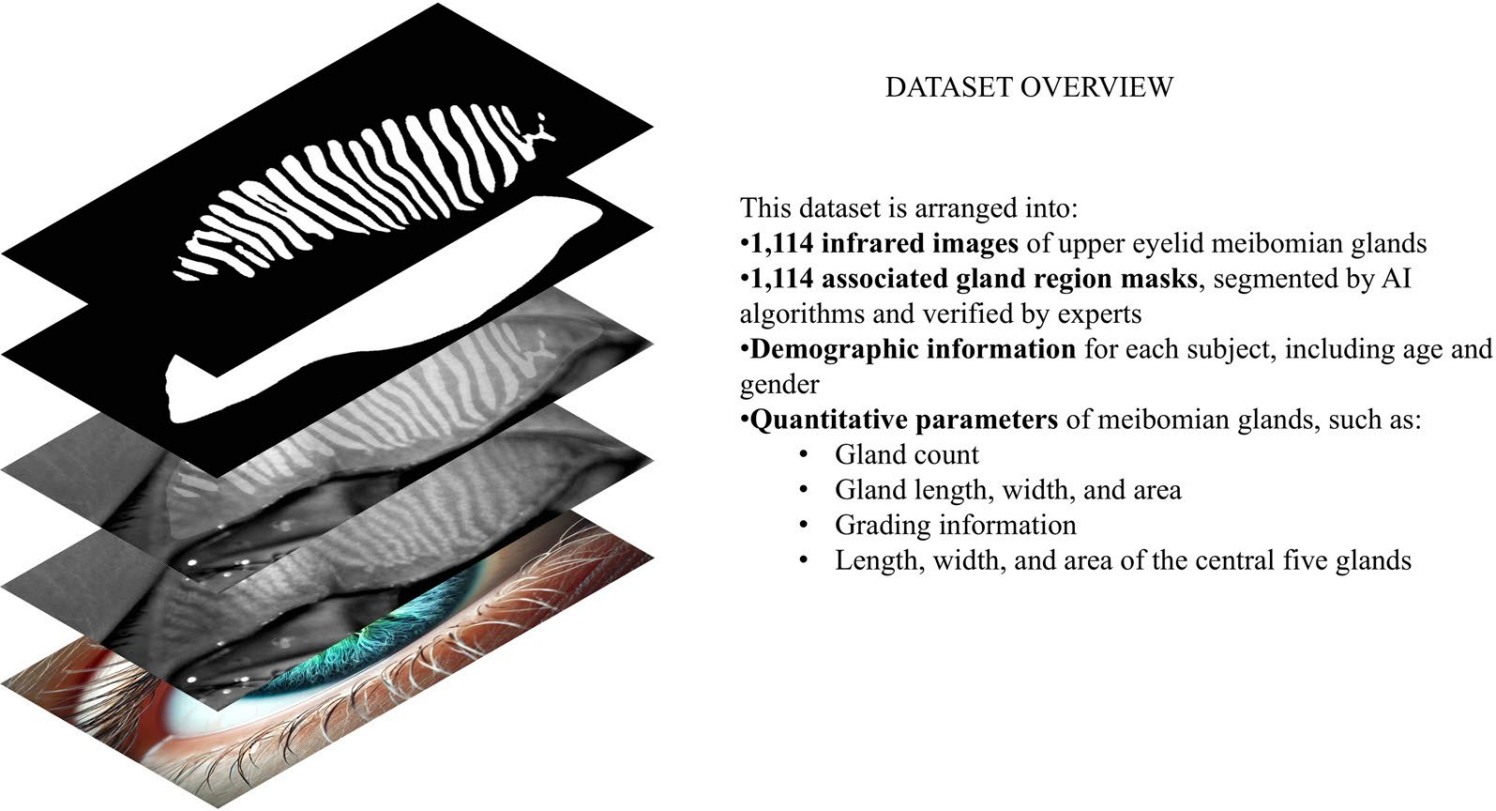
Overview of the Children and Adolescents Meibomian Gland (CAMG) dataset. The CAMG dataset consists of a comprehensive collection of 1114 infrared images of upper eyelid meibomian glands from children and adolescents (ages 4–18 years). All images were carefully annotated using intelligent algorithms and subsequently corrected by expert ophthalmologists to ensure high-quality segmentation for accurate analysis

Initially, a total of 1563 infrared images of MGs were captured between June 2020 and July 2024. After preliminary screening, 363 images were excluded due to duplicate captures, incomplete eyelid eversion, poor tarsal exposure, or positioning errors, resulting in 1200 candidate images derived from 730 participants (with one upper eyelid image per eye). Subsequently, 86 images were excluded due to reflections and blurring of peripheral gland structures. Consequently, 1114 high-quality upper eyelid images were included in the final CAMG dataset (Fig. 1). To enhance gland visibility and improve the performance of downstream AI segmentation, all images used for this dataset were contrast-enhanced versions of the original infrared images.

The project was approved by the Ethics Committee of Fujian Provincial Hospital (Approval No. K202003124), and all procedures adhered to the ethical standards set forth in the Declaration of Helsinki for human research. Informed consent was obtained from the guardians of all participants.

### Details of CAMG dataset

The CAMG dataset, available in the public Figshare repository (10.6084/m9.figshare.27601101.v7) provides a comprehensive resource for research in MG health (Table 1). It comprises 1114 high-quality upper eyelid meibography images collected from 730 participants aged 4–18 years (mean age: 11.80 ± 2.39 years), with a slight female predominance (53.23%). All images were captured using the Oculus Keratograph 5 M by a trained technician under quality-controlled imaging conditions. The dataset includes grayscale infrared images, and the image acquisition was conducted over a four-year period from June 2020 to July 2024. All participants were of Han ethnicity and were recruited from Fujian Provincial Hospital in Southeastern China. Following the WHO’s definitions, participants aged 4–17 years were classified as children and those aged 10–18 years as adolescents, with partial overlap between groups. A total of 1107 images were categorized into the children cohort, with a mean age of 11.76 ± 2.34 years and a sex distribution of 518 males (46.79%) and 589 females (53.21%). The adolescent cohort included 939 images, with a mean age of 12.45 ± 1.97 years, comprising 431 males (45.90%) and 508 females （54.10%）.

**Table 1:** Children and Adolescents Meibomian Gland (CAMG) dataset demographic information

Characteristics	Values
Number of subjects	730
Age distribution range (years)	4–18
Average age (years)	11.80 ± 2.39
Sex (based on images)	
Male	521 (46.77%)
Female	593 (53.23%)
Total number of MG images	1114
Color channel	Single/Grayscale
Imaging device	Keratograph 5 M (Oculus, Germany)
Duration of data collection	June 2020 to July 2024 (4 years)
Ethnicity of subjects Recruitment location	Han ethnicity Fujian provincial hospital in Southeastern China
Children cohort (4–17 years)	
Images	1107
Average age (years)	11.76 ± 2.34
Sex (based on images)	
Male	518 (46.79%)
Female	589 (53.21%)
Adolescents cohort (10–18 years)	
Images	939
Average age (years)	12.45 ± 1.97
Sex (based on images)	
Male	431 (45.90%)
Female	508 (54.10%)

Here, we provide one hundred photographs for reference, with additional photos available upon request from the corresponding author through email. The main file, "CAMG dataset.xlsx", includes participants’ demographic information, such as age and gender, as well as MG parameters, including average gland length, width, area, central gland length, width, area, and gland count. Anonymized MG images, captured at a resolution of 888 × 414, are stored in the “img” folder. Segmented images for meibomian area and gland segmentation are found in the “roi” and “seg” folders, respectively, with segmentation performed using the U-Net model. Seg and ROI images were proportionally scaled down before undergoing segmentation. Detailed MG parameter data is stored in CSV format in the “csv” folder for statistical analysis and model training. Since each subject contributed two eyes, we first evaluated the correlations between left and right eyes for all gland parameters. Spearman’s correlation coefficients were calculated, which demonstrated generally low correlations (rs = 0.03–0.64, Supplementary Table 1). Considering this modest level of between-eye dependence, we primarily treated eyes as independent observations.

In addition, we compared the segmentation performance of several widely used deep learning models on the previous dataset [25]. Following the setup of previous studies [25, 26], the dataset was split into training, validation, and test sets in a 7:1:2 ratio, and five-fold cross-validation was employed to obtain stable average results. To ensure a fair comparison, all models were trained and evaluated using identical experimental parameter settings. During model training, the random cropping strategy involved selecting different regions of interest within the meibomian gland images to introduce variability in the dataset. The batch size was set to 8, the total number of epochs was set to 150, and the Adam optimizer was used to optimize the model with an initial learning rate of 0.0003. To prevent overfitting, a learning rate adjustment strategy was adopted, reducing the learning rate to half every 50 epochs. We evaluated the performance of the segmentation models using intersection over union (IoU), Dice coefficient, and accuracy (ACC). The experimental model achieved an accuracy of 97.49%, demonstrating the high precision of the segmentation. The Dice coefficient was 89.72%, indicating a significant overlap between the predicted segmentation and the ground truth. The IoU score reached 81.67%, reflecting the model's effectiveness in capturing the intersection between the predicted and actual regions, minimizing false positives and false negatives. Details for comparison with other models can be found in Table 2. The code for processing and analyzing the dataset, along with the trained U-Net model weights (available via Google Drive link), is accessible on GitHub (https://github.com/ljw-fzu/AI_for_CAMG) This ensures that readers can readily reproduce and build upon our results.

**Table 2:** Performance comparison of deep learning models for meibomian gland (MG) segmentation

Algorithms	IoU/%	Dice	ACC
UNet [27]	81.67	89.72	97.49
UNet++ [53]	78.85	87.90	97.38
UNet3+ [28]	75.39	85.55	96.90
Attention-UNet [54]	79.64	88.40	97.47
HRNet [55]	79.24	88.16	97.43
DenseUNet [56]	79.61	88.39	97.49
Swin-UNet [29]	39.50	55.87	83.73
CE-Net [57]	80.96	89.30	97.40
MNet [58]	37.69	54.30	87.37
UNet3 [28]	80.88	89.22	97.39

Models were evaluated on identical datasets using IoU, Dice coefficient (spatial overlap), and accuracy metrics

*IoU* = intersection over union; *ACC* = accuracy

### Image processing and quality control

All meibography images were captured using the Keratograph 5 M under quality-controlled infrared illumination. To minimize specular reflections, technicians optimized the eyelid eversion technique and adjusted participant positioning relative to the device’s light source. Consistent ambient lighting was maintained throughout.

The collected infrared meibography images, acquired using the Keratograph 5 M, were preprocessed to enhance image quality and ensure consistency across the dataset. This preprocessing primarily involved two operations: image denoising and normalization. First, the built-in glare reduction function of the Keratograph 5 M was utilized to minimize specular reflections while preserving the structural and edge features of the MGs. Subsequently, all images were uniformly resized to a resolution of 888 × 414 and normalized to minimize intensity variations caused by lighting differences during image acquisition.

**Fig. 2:**
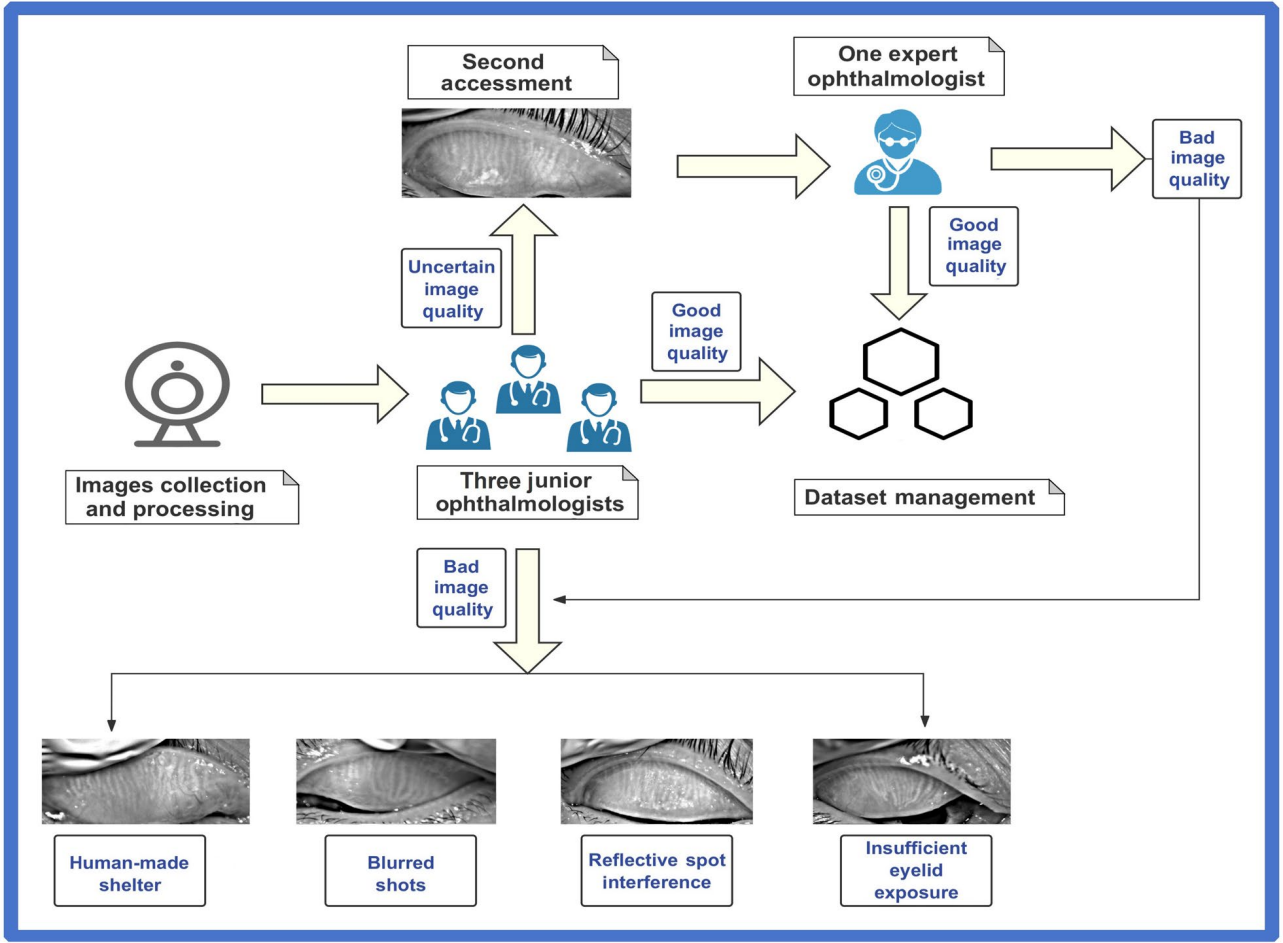
Workflow of meibomian gland (MG) infrared image screening

For image screening (Fig. 2), all preprocessed images were evaluated by three independent ophthalmologists to determine whether they met the predefined quality standards. These standards included image clarity, focus, and the visibility of key anatomical structures, such as the MGs and tarsal plates. Images were excluded if they contained more than three specular reflection spots and if any single spot markedly interfered with the segmentation or assessment of the MGs. In cases where the three ophthalmologists disagreed on the quality assessment, the image was forwarded to a senior ophthalmologist with over 10 years of experience in MG imaging for final adjudication. This rigorous quality control process ensured that only high-quality images were included in the subsequent analysis, thereby enhancing the reliability and validity of the study results.

### Generation of morphological parameters

**Fig. 3:**
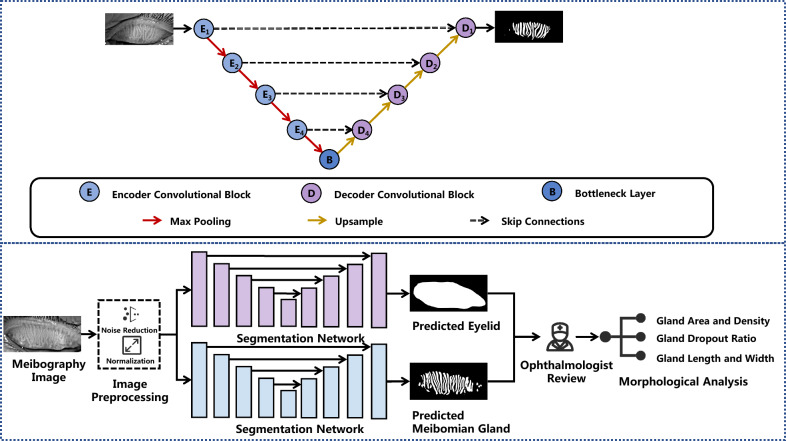
Deep learning framework for automated meibomian gland (MG) analysis. U-Net architecture with encoder pathway (E1–E4) for feature extraction, decoder pathway (D1–D4) for reconstruction, and skip connections (dashed lines) preserving spatial details. Complete workflow: meibography images undergo preprocessing and dual-network segmentation to predict eyelid and MG masks, enabling automated morphological analysis for clinical assessment

**Fig. 4:**
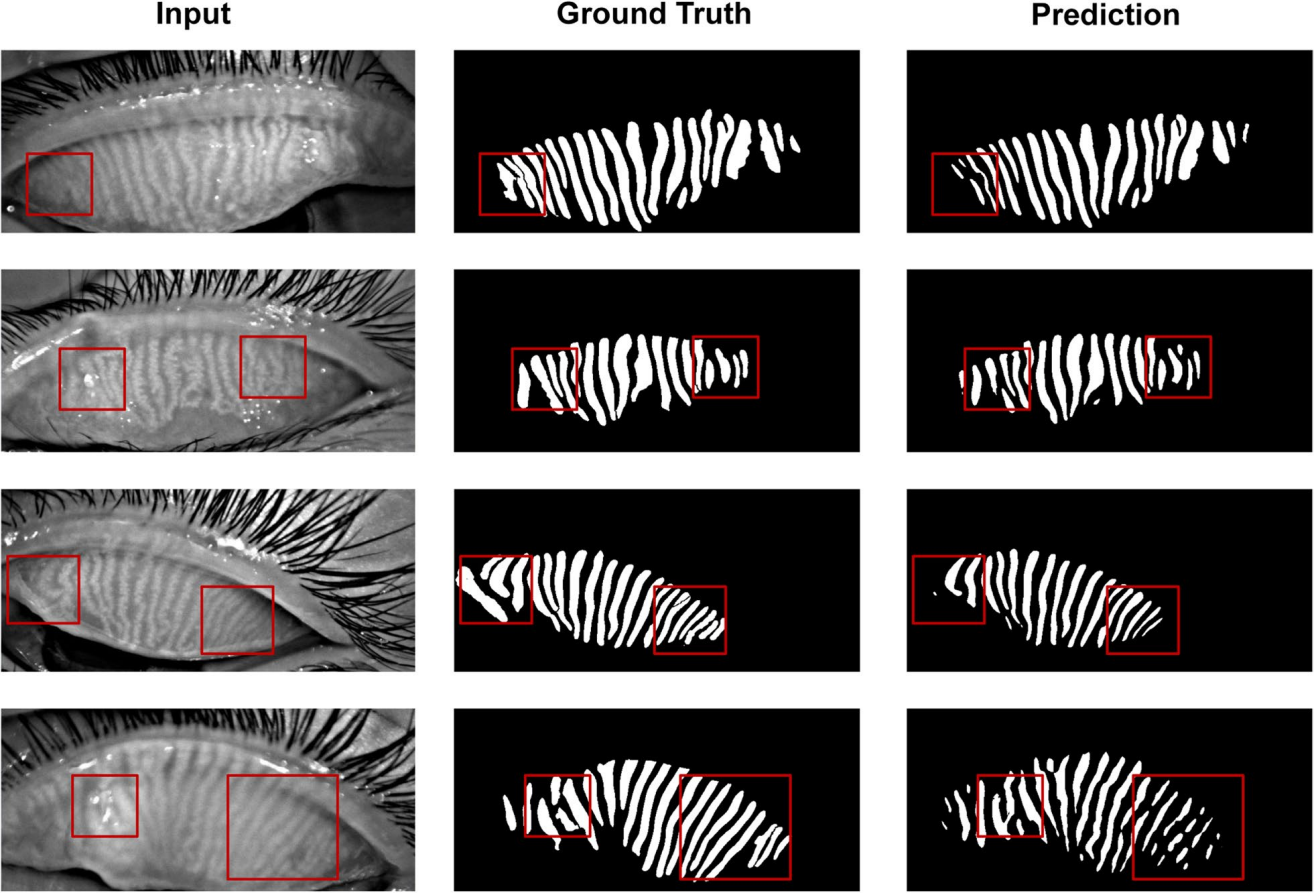
Visualization of U-Net segmentation results on the training dataset. The model performs poorly when handling blurry edges and areas with strong reflections

U-Net is a lightweight yet powerful segmentation network that employs a symmetrical encoder-decoder structure, utilizing skip connections to fuse shallow and deep features for better feature representation. It is widely used in medical imaging [27–30]. Therefore, we applied U-Net for automatic segmentation of MG images (Fig. 3). Specifically, we first preprocessed the collected infrared MG images to improve image quality, including denoising, normalization, and contrast enhancement. Then, the input image is fed into the encoder, where multiple convolution and max pooling operations are used to extract deeper features and reduce the size. The encoder’s output feature maps at the bottleneck layer capture the most abstract features. Subsequently, in the decoder part, the feature maps are progressively sampled to recover the size, and skip connections are used to concatenate the encoder’s and decoder’s feature maps to retain detailed information. Finally, the model generates segmentation results through the output layer. This U-Net model was previously developed and validated in our earlier work [25], where it achieved high segmentation accuracy (94.09%) and strong agreement with manual annotations from multiple ophthalmologists (Kappa = 0.93). External validation across four ophthalmic centers including Zhongshan Ophthalmic Center, Dongguan Huaxia Eye Hospital, Zhuzhou City Hospital, and Putian Ophthalmic Hospital, further demonstrated its robustness and generalizability. In this study, we applied this validated model under expert supervision to generate automated segmentations and extract quantitative morphological parameters for the CAMG dataset. Figure 4 illustrates some failure cases and edge scenarios of the U-Net model applied to the previous dataset. It can be observed that while the model performs well in capturing the overall morphology of glands in the central region, its performance is suboptimal when dealing with blurry edges and strong reflection areas. Therefore, during the construction of the CAMG dataset, ophthalmology experts paid special attention to these critical regions and performed meticulous corrections.

During model training, the random cropping strategy involved selecting different regions of interest within the MG images to introduce variability in the dataset. The batch size was set to 8, the total number of epochs was set to 150, and the Adam optimizer was used to optimize the model with an initial learning rate of 0.0003. To prevent overfitting, a learning rate adjustment strategy was adopted, reducing the learning rate to half every 50 epochs.

The final generated parameters are as follows: (1) Gland area: the total number of pixels occupied by the glands in the gland masks; (2) Gland dropout ratio: the proportion of missing glands relative to the total area of the MGs; (3) Gland length and width: the length of the gland's central line and the length of the perpendicular bisector of this central line are defined as the gland's length and width, respectively; (4) Number of glands: the number of glands per unit area of the eyelid mask; (5) Total glands ratio: the proportion of the total area occupied by glands relative to the entire eyelid area. The total gland ratio and gland dropout ratio are complementary and sum to 1, as they together represent the full proportion of the tarsal area. For each image, the five central meibomian glands were selected based on the total number of segmented glands (n). If n was odd, (n − 5)/2 glands were removed from both the nasal and temporal sides. If n was even, (n − 6)/2 glands were removed from the nasal side and (n − 4)/2 from the temporal side, ensuring that the five remaining glands were located centrally.

Considering the important role of the central five MGs in lipid secretion and their easier recognition by the algorithm [31], we extracted the corresponding data from these glands.

### Statistical analysis

Descriptive statistics are presented as mean ± standard deviation (SD) for continuous variables and count (percentage) for categorical variables. The Shapiro–Wilk test was used to assess the normality of the data distribution. For comparisons between two groups, the t-test was applied when both datasets followed a normal distribution; otherwise, the Mann–Whitney U test was used. For comparisons across age groups, one-way analysis of variance (ANOVA) was employed for normally distributed data, while the Kruskal–Wallis H test was used for non-normally distributed data, followed by Dunn’s test for post hoc analysis. Line plots were used to visualize trends in glandular parameters with age, and forest plots were used to illustrate the distribution of central glands in children and adolescents. A two-sided *P* value of less than 0.05 indicates statistical significance. All analyses were performed using R software version 4.4.1 (The R Foundation for Statistical Computing).

## Results

### MG morphology parameter in children and adolescents

**Fig. 5:**
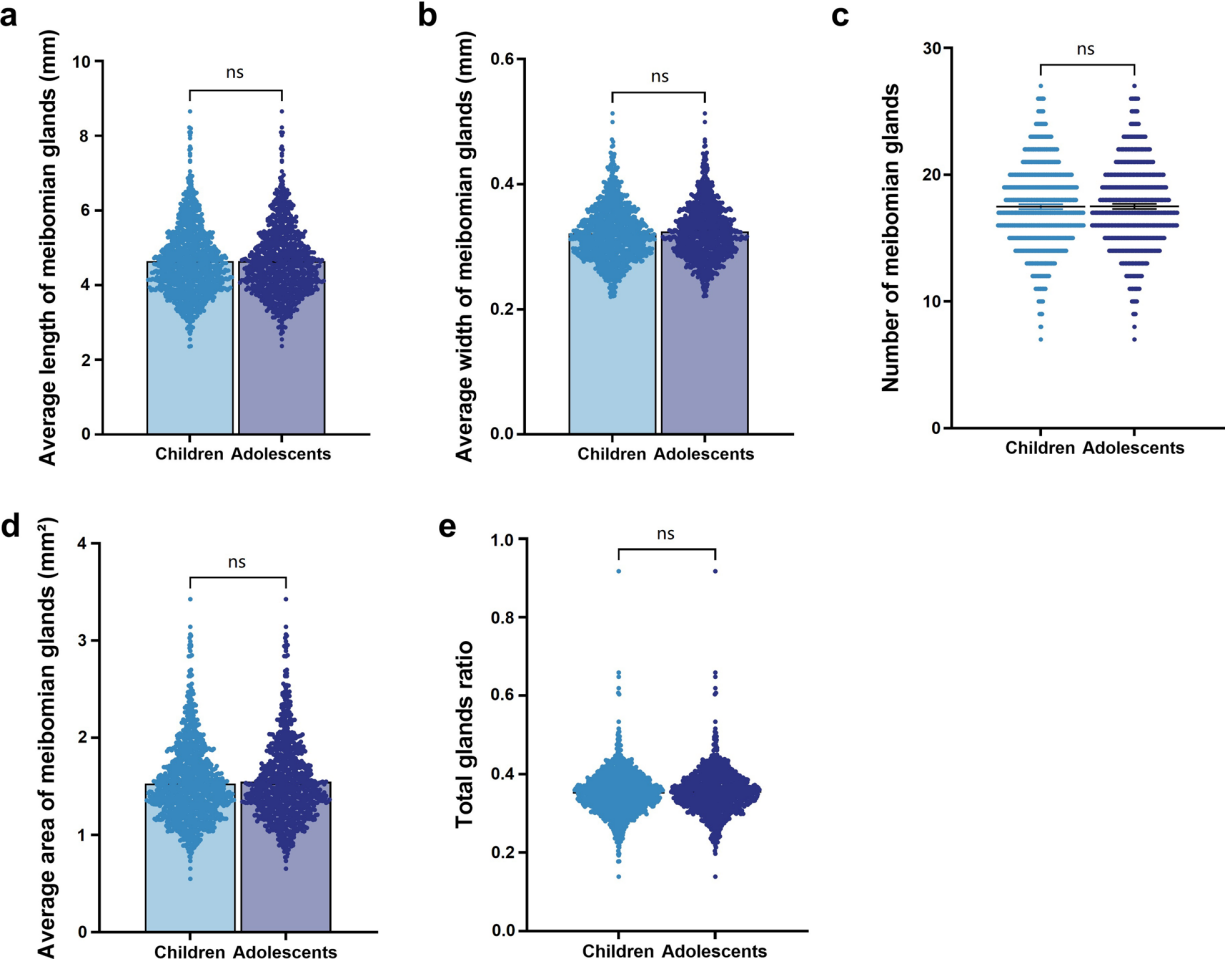
Benchmark data of meibomian gland (MG) parameters in children and adolescents.** a** Average length of MGs. **b** Average width of MGs. **c** Number of MGs per individual. **d** Average area of MGs. **e** Total gland ratio, representing the proportion of the eyelid occupied by glands

The average gland length was 4.643 mm (95% CI: 4.589–4.697 mm) in children and 4.647 mm (95% CI: 4.589–4.706 mm) in adolescents (Fig. 5a). The average width and area were 0.321 mm (95% CI: 0.319–0.324 mm) and 1.531 mm^2^ (95% CI: 1.508–1.555 mm^2^) in children, compared to 0.324 mm (95% CI: 0.321–0.327 mm) and 1.549 mm^2^ (95% CI: 1.523–1.575 mm^2^) in adolescents (Fig. 5b, d). The mean number of glands was 17.48 (95% CI: 17.29–18.67) in children and 17.50 (95% CI: 17.29–17.71) in adolescents (Fig. 5c). Similarly, the total glands ratio remained stable between the two groups (0.353 and 0.355; Fig. 5e). No significant differences were observed in MG parameters between the child group and the adolescent group (all *P*s > 0.05).

**Fig. 6:**
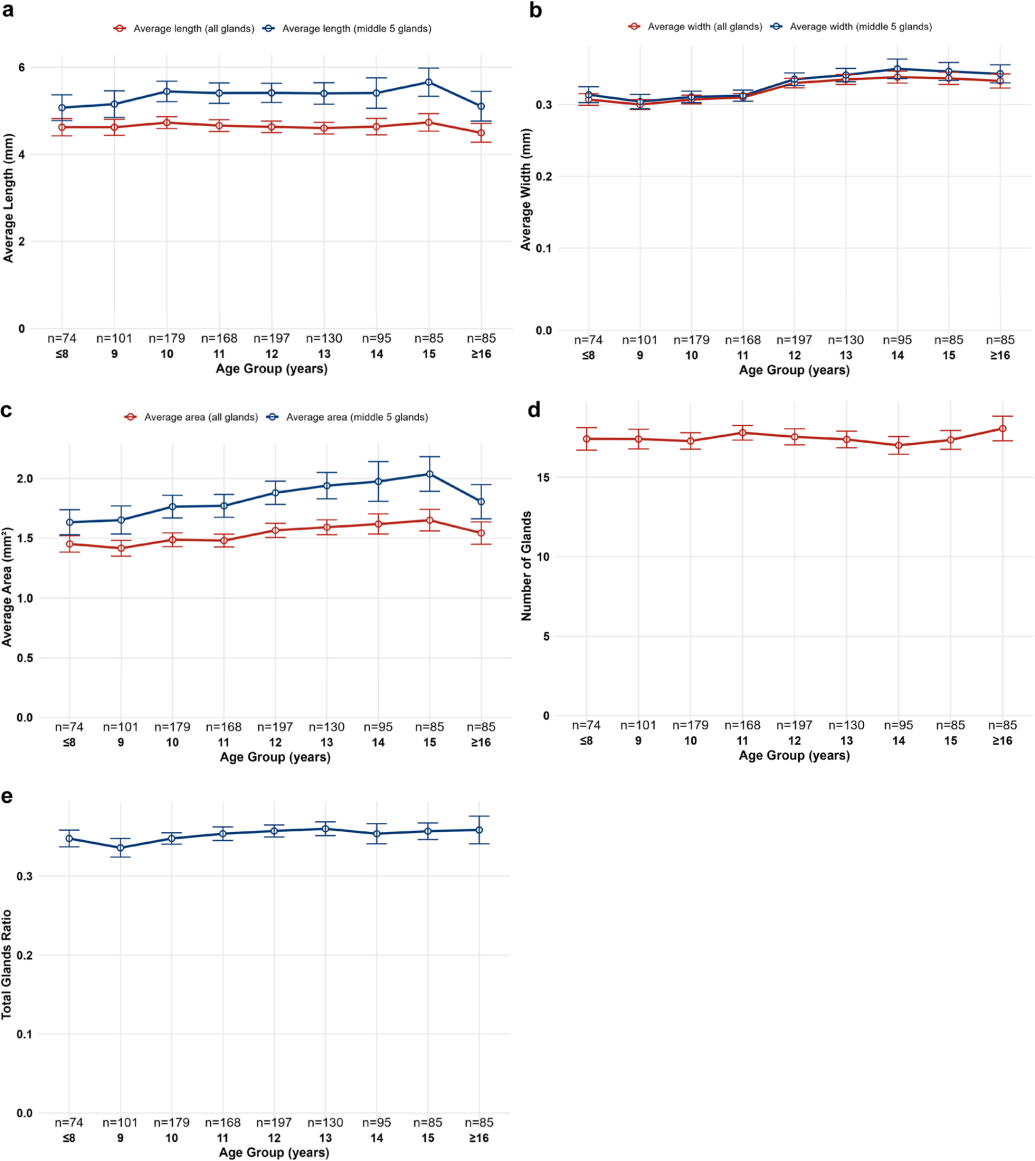
Age-related changes in meibomian gland (MG) morphological parameters.** a** Average gland length across age groups, comparing all glands (red line) and the middle five central glands (blue line). **b** Average gland width, comparing all glands (red line) and the middle five central glands (blue line). **c** Average gland area,, comparing all glands (red line) and the middle five central glands (blue line). **d** Number of visible MGs across age groups. **e** Total gland ratio across age groups. Data are presented as mean ± 95% CI

Further analysis across more detailed age subgroups (Fig. 6a–e) showed that MG parameters remained largely stable with increasing age, without significant variations observed (all *P*s > 0.05).

### Gender differences in MG morphology parameters in children and adolescents

Among children, females exhibited significantly greater MG width (0.325 mm, 95% CI: 0.322–0.328 mm) and area (1.542 mm^2^, 95% CI: 1.512–1.572 mm^2^) compared to males (0.317 mm, 95% CI: 0.313–0.321 mm; 1.519 mm^2^, 95% CI: 1.483–1.556 mm^2^, respectively; *P* < 0.05). In the adolescent group, similar trends were observed, with females showing significantly greater gland width (0.328 mm, 95% CI: 0.324–0.331 mm) than males (0.321 mm, 95% CI: 0.316–0.325 mm; *P* < 0.05).

**Fig. 7:**
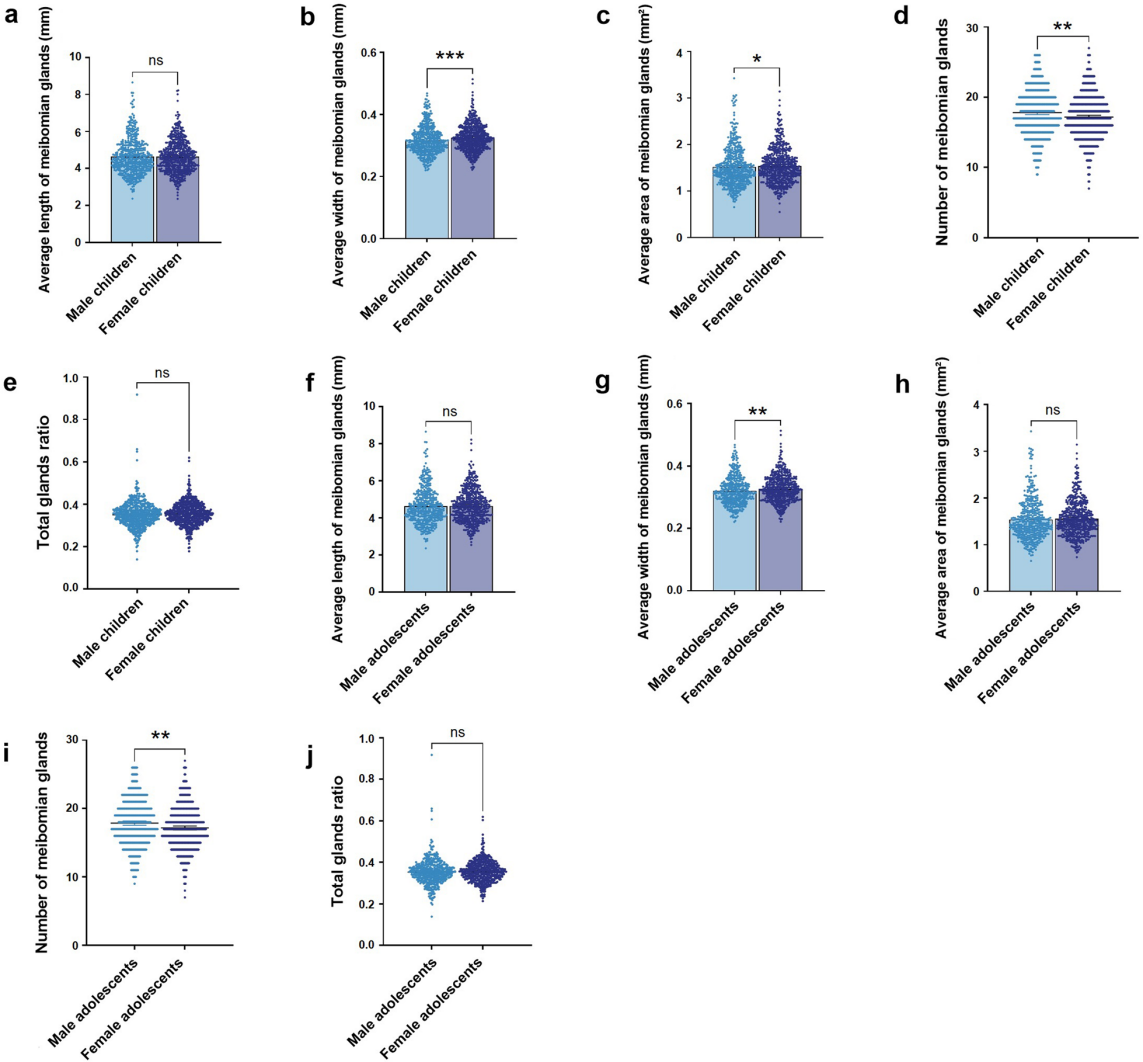
Sex-related differences in meibomian gland (MG) morphological parameters among children (**a–e**) and adolescents (**f–j**). **a, f** Average length of MGs. **b, g** Average width of MGs. **c, h** Average area of MGs. **d, i** Number of visible MGs. **e, j** Total glands ratio

However, male children (17.81, 95% CI: 17.53–18.10) and adolescents (17.88, 95% CI: 17.56–18.20) exhibited a higher MG count compared to females (17.18, 95% CI: 16.92–17.44; 17.17, 95% CI: 16.90–17.45; *P* < 0.05). No significant sex-related differences were observed in other parameters. More detailed distributions are presented in Fig. 7.

### Middle glands differences in MG morphology parameters in children and adolescents

**Fig. 8:**
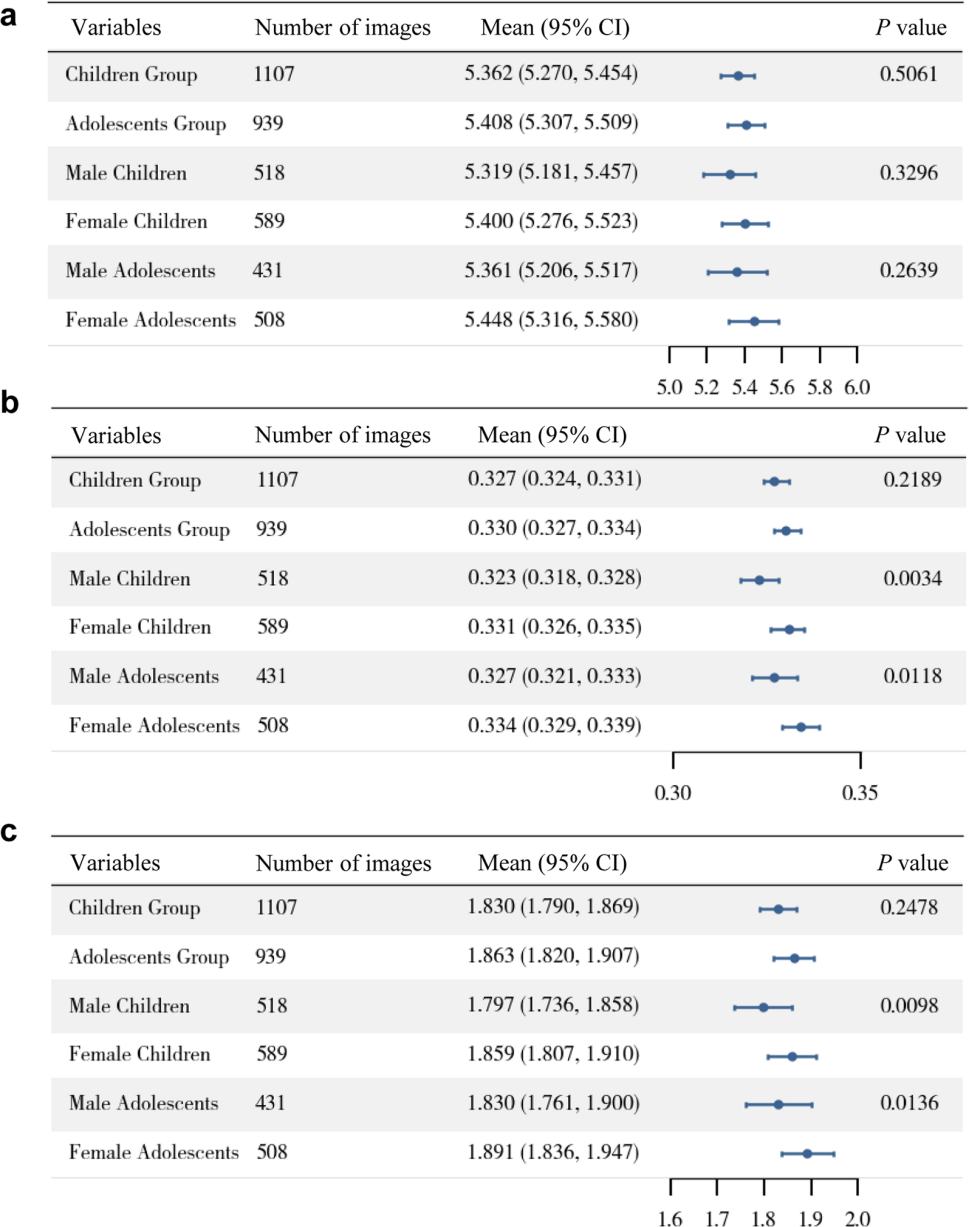
Forest plots comparing morphological parameters of the central five meibomian glands (MGs) across different age and sex groups.** a** Average gland length. **b** Average gland width. **c** Average gland area. The numbers (1107 for children and 939 for adolescents) refer to infrared eyelid images, not individual participants. Following the WHO definitions, participants aged 4–17 years were classified as children, and those aged 10–18 years as adolescents. As these age groups partially overlap, some images fall into both categories. Therefore, the total number of images in the two groups do not add up to 1,144

As illustrated in Fig. 8, significant sex-related differences were observed in both gland width and area. Female children exhibited a greater average width (0.331 mm, 95% CI: 0.326–0.335 mm) than male children (0.323 mm, 95% CI: 0.318–0.328 mm), with a statistically significant difference (*P* = 0.0034). Similarly, female adolescents demonstrated a wider MG (0.334 mm, 95% CI: 0.329–0.339 mm) compared to male adolescents (0.327 mm, 95% CI: 0.321–0.333 mm; *P* = 0.0118). Female children also showed a significantly larger area (1.859 mm^2^, 95% CI: 1.807–1.910 mm^2^) than male children (1.797 mm^2^, 95% CI: 1.736–1.858 mm^2^; *P* = 0.0098). A similar trend was observed in adolescents, where female participants had greater MG area (1.891 mm^2^, 95% CI: 1.836–1.947 mm^2^) than their male counterparts (1.830 mm^2^, 95% CI: 1.761–1.900 mm^2^; *P* = 0.0136).

## Discussion

This study provides the first open-access benchmark dataset dedicated to children and adolescent populations. Comprising 1114 quality-controlled infrared meibography images from 730 participants, the dataset integrates rigorous preprocessing, and AI-driven segmentation using a U-Net model. We specifically focused on the upper eyelid MGs as they constitute approximately 61% of the total gland count and are more prone to morphological abnormalities such as distortion, hook-like shapes, and overlap [22, 32]. Moreover, most dry eye symptoms and clinical signs are significantly associated with upper eyelid MG morphology [33]. Key morphological parameters—including gland length, width, area, ratio, and age- and gender-stratified reference values—were quantified. The segmentation model achieved high accuracy (97.49%) and moderate overlap metrics (Dice: 89.72%; IoU: 81.67%), demonstrating its utility for automated analysis.

Our dataset explicitly documents quality control steps—from hardware standardization to multi-stage expert reviews—setting a new standard for transparency. In contrast, previous datasets often lacked transparent image exclusion criteria or quality control workflows, raising concerns about hidden biases [34–36]. For instance, Wang et al. [12] relied on manual annotation for segmentation but did not provide detailed information about inter-annotator agreement, whereas CAMG’s structured annotation protocol ensures reproducibility. This ensures the reliability of the dataset used for algorithm training.

Our findings indicate that MG parameters including length (4.643–4.647 mm), width (0.321–0.324 mm), area (1.531–1.549 mm2), gland count (17.48–17.50), and total gland ratio (0.353–0.355) remain relatively stable across different age groups of children and adolescents, suggesting that MG morphology may reach a developmental plateau during this stage of life. This observation aligns with previous reports demonstrating that MGs undergo rapid development early in life and then maintain consistent morphological features throughout later childhood and adolescence [37]. It has been proposed that gland maturation and structural stability might be achieved early, after which gland morphology remains less influenced by age-related changes until adulthood [3, 38].

Interestingly, our study found that females exhibited significantly greater MG width and area compared to males across both children and adolescents. Specifically, gland width in females was 0.325 mm (95% CI: 0.322–0.328 mm) and 0.328 mm (95% CI: 0.324–0.331 mm), while in males it was 0.317 mm (95% CI: 0.313–0.321 mm) and 0.321 mm (95% CI: 0.316–0.325 mm). In terms of gland area among children, females showed 1.542 mm^2^ (95% CI: 1.512–1.572 mm^2^) compared to 1.519 mm^2^ in males (95% CI: 1.483–1.556 mm^2^). Although these differences were statistically significant, the effect sizes were modest, and their clinical relevance remains uncertain. Given the relatively small magnitude of these sex-related differences, we advise cautious interpretation and recommend that future studies incorporate longitudinal or functional assessments (e.g., lipid quality, tear film stability) to further clarify their clinical implications. This sex-related difference may primarily be attributed to hormonal influences, particularly estrogen and progesterone, which are more prevalent in females and have been reported to impact sebaceous gland activity and morphology [39]. Estrogen receptors have been identified in human MGs, suggesting direct hormonal regulation of glandular differentiation, lipid secretion, and possibly glandular hypertrophy [40]. Meanwhile, most epidemiological studies suggest that males are more prone to MGD than females [41, 42], and glandular parameters are predictive indicators of MGD [43]. Our observations may help explain why males could be more susceptible to MGD. Considering the critical role of the middle five MGs in lipid secretion and their typically stable imaging quality, we conducted a focused analysis on these glands [44, 45]. Consistently, we observed similar findings, which further validated our conclusions. In addition, we found that male children (17.81, 95% CI: 17.53–18.10) and adolescents (17.88, 95% CI: 17.56–18.20) exhibited a higher MG count compared to females (17.18, 95% CI: 16.92–17.44; 17.17, 95% CI: 16.90–17.45). We hypothesize that the marked increase in androgen levels during puberty not only stimulates the proliferation and differentiation of MG epithelial cells but also accelerates the formation and maturation of new glands, resulting in a significantly higher number of MGs in males compared with females [46, 59].

In contrast to earlier studies that primarily used semi-quantitative grading or simple gland counts, our study employed direct measurements of gland dimensions. This methodological improvement allows for the detection of subtle morphological changes that grading scales might overlook [3]. Identifying deviations from the expected gland length or area for a given age can facilitate early intervention. For instance, a recent study demonstrated that children with allergic conjunctivitis have significantly shorter glands and smaller glandular areas compared to healthy peers [47]. In this research context, our reference dataset serves as a foundation for future investigations into how environmental factors, hormonal influences, or disease states may affect MG development in the young population.

Prior datasets, such as MGD-1K [11], predominantly focus on adults (mean age: 54 years), limiting their relevance to pediatric populations. In contrast, CAMG’s emphasis on younger age groups aligns with rising concerns about early-onset MG dysfunction linked to environmental factors and screen time. On the other hand, the MGD-1 K dataset relies on LipiView imaging, whereas our dataset utilizes the Keratograph 5 M device, together providing a complementary source of data from diverse imaging technologies that enhances the robustness and applicability of MG research. While U-Net architectures have been widely used for adult meibography [12, 13, 48, 49], this study demonstrates their adaptability to pediatric images, achieving comparable performance to models trained on adult-centric data. Additionally, the inclusion of demographic data enhances the dataset’s utility for epidemiological studies, a feature that was absent in earlier datasets. Furthermore, although previous studies [4, 11, 50] claimed to provide both upper and lower eyelid images, the number of upper eyelid images in those datasets may be limited due to the difficulty in exposing the upper eyelid, which requires eversion that may cause discomfort to the patient [51], making it more difficult for them to cooperate. Our study specifically provides a large number of upper eyelid images of children, thereby offering richer data for related analyses. Quality control protocols further distinguish CAMG from existing datasets: while previous datasets often lack standardized quality assessment, CAMG implements rigorous multi-level quality control including image resolution verification, anatomical landmark validation, and annotation consistency checks performed by experienced ophthalmologists.

Clinically, CAMG provides reference values to guide individualized assessments, enhancing diagnostic accuracy in pediatric dry eye disease. Numerous studies have found a relatively high incidence of MG atrophy in children. Emerging evidence underscores the concerning prevalence of MG atrophy in this population. For example, one study reported that 42% of 99 children exhibited signs of MG atrophy [20], while another study noted that 31% of participants showed severe atrophy in at least one eyelid [52]. However, it remains unclear which specific morphological patterns of types of atrophy such as gland shortening, dropout or reduced area are clinically significant. The CAMG dataset addresses this gap by providing age- and gender-stratified normative parameters, allowing clinicians to differentiate between physiological variations and pathological atrophy with unprecedented precision.

While this study provides a valuable open-access dataset (CAMG) for MG analysis in pediatric populations, several limitations should be acknowledged. First, the CAMG dataset only includes participants from southeastern coastal China. Future multi-center studies across diverse regions are needed to enhance generalizability. Second, the dataset contains only upper eyelid MG images. While this focus was justified by the upper eyelid's predominant gland distribution (61% of total glands) and stronger clinical correlations with dry eye symptoms, the exclusion of lower eyelid assessment limits comprehensive MG evaluation. Technical challenges in obtaining high-quality lower eyelid images in pediatric populations necessitate future imaging protocol optimizations to enable complete eyelid assessments. Third, all quantitative analyses were performed using a single in-house AI model. Applying additional validated algorithms to this dataset could further verify the consistency of morphological metrics. Fourth, the current hospital-based participant recruitment pattern may not comprehensively reflect the overall characteristics of the pediatric population, and the dataset lacks multi-ethnic representation, which could limit the applicability of findings across different racial and ethnic groups. Fifth, while the selection of the central five glands was standardized based on the total number of segmented glands, we did not specifically evaluate the repeatability of central gland identification across repeated analyses. Instead, our validation focused on the overall segmentation accuracy and repeatability of all meibomian glands, which have been comprehensively assessed in our previous study [25]. Future studies should extend this evaluation to the central five glands to further enhance reliability. Sixth, lifestyle-related information was not collected in this study, which limits our ability to assess the potential influence of such factors on meibomian gland morphology. For example, we could not explore whether height is correlated with gland length or width. Future studies could consider prospective follow-up, such as re-examining participants after five years to compare gland parameters following growth in height, thereby providing valuable longitudinal insights. Finally, we did not collect data on ocular surface health status or subjective symptoms such as dry eye questionnaires (e.g., OSDI or SPEED), limiting our ability to correlate morphological features with clinical manifestations. Future studies should incorporate both structural and functional assessments to improve the translational value and generalizability of the dataset.

## Conclusion

The CAMG dataset bridges a vital gap in ocular surface research by offering a quality-controlled, pediatric-centric resource for AI algorithm training and clinical benchmarking. Its rigorous annotation pipeline, demographic stratification, and open accessibility position it as a cornerstone for advancing precision diagnostics in pediatric dry eye disease. We anticipate that this dataset will catalyze global collaborations to decode the developmental trajectories of MGs and accelerate AI innovations tailored to young populations.

## Supplementary Information

Additional file 1.

## Data Availability

The CAMG dataset is publicly available in the Figshare repository at (10.6084/m9.figshare.27601101.v7). The accompanying source code for image processing, quantitative analysis, and visualization is openly accessible on GitHub (https://github.com/ljw-fzu/AI_for_CAMG).
