# Supplementary material for: AI driven quantitative analysis of meibomian glands in children and adolescents: a benchmark dataset study
Source: Eye Vis (Lond). 2025 Nov 6;12:46. doi: 10.1186/s40662-025-00460-2 (PMC12590672; doi:10.1186/s40662-025-00460-2)
Supplement: Supplementary file 1 — Additional file 1. [file 40662_2025_460_MOESM1_ESM.docx]

**Supplementary Table 1.** Median interquartile range (IQR) of meibomian gland parameters in left and right eyes and their correlations.

| Parameter | Left eye median (IQR) | Right eye median (IQR) | Spearman’s R |
| --- | --- | --- | --- |
| Number of glands | 17 (15, 20) | 15 (17, 19) | 0.23 |
| Total glands ratio | 0.35 (0.32, 0.40) | 0.37 (0.34, 0.41) | 0.47 |
| Length | 4.35 (3.89, 5.09) | 4.77 (4.16, 5.45) | 0.27 |
| Width | 0.32 (0.29, 0.34) | 0.32 (0.30, 0.35) | 0.64 |
| Area | 1.42 (1.21, 1.71) | 1.55 (1.34, 1.85) | 0.38 |
| Curvature | 12.68 (5.43, 26.95) | 16.30 (7.14, 31.37) | 0.03 |
